# Self-Antigen Presentation by Dendritic Cells in Autoimmunity

**DOI:** 10.3389/fimmu.2014.00055

**Published:** 2014-02-13

**Authors:** Ann-Katrin Hopp, Anne Rupp, Veronika Lukacs-Kornek

**Affiliations:** ^1^Department of Medicine II, Saarland University Medical Center, Homburg, Germany

**Keywords:** self-antigen presentation, peripheral tolerance, tolerogenic dendritic cells, dendritic cell subtypes and autoimmunity

## Abstract

The operation of both central and peripheral tolerance ensures the prevention of autoimmune diseases. The maintenance of peripheral tolerance requires self-antigen presentation by professional antigen presenting cells (APCs). Dendritic cells (DCs) are considered as major APCs involved in this process. The current review discusses the role of DCs in autoimmune diseases, the various factors involved in the induction and maintenance of tolerogenic DC phenotype, and pinpoints their therapeutic capacity as well as potential novel targets for future clinical studies.

## Introduction

Immune reaction against self-antigens is primarily prevented within the thymus in a process called central tolerance (1). Despite the rigorous screening of the evolving T-cell repertoire, some autoreactive T cells escape from the thymus (1). To avoid autoimmunity, multiple operations ensure the control of the “escaped” T-cell repertoire at the periphery such as induction of anergy, deletion of autoreactive T cells, and activation or induction of regulatory T cells (Tregs) (2, 3). The presentation of self-antigens at the periphery, similarly to the thymus, is carried out by multiple antigen presenting cells (APCs) such as stromal cells and dendritic cells (DCs) (4). This review focuses on DCs as principal APCs involved in this process.

Dendritic cells are present in all tissues and involved in the initiation of immune responses (5). They are capable of recognizing pathogens and various danger signals, which leads to the upregulation of their co-stimulatory molecules, production of cytokines, and activation and effector differentiation of pathogen-specific T cells. Additionally, via communicating with various immune cells [e.g., natural killer cells (NKs), natural killer T (NKT) cells] they bridge the innate and adaptive arm of the immune response (5).

Dendritic cells are a heterogeneous cell population consisting of multiple subtypes (6). Major populations of DCs present in murine secondary lymphoid organs (SLOs) are CD8^+^, CD8^−^ DCs, and plasmacytoid DCs (pDCs). The CD8^−^ DCs can be further subdivided into three groups: CD4^+^, double negative (DN) (CD11c^+^ CD11b^+^ CD4^−^ CD8^−^), and triple negative (TN) subset (CD11c^+^ CD11b^−^ CD4^−^ CD8^−^) (Table 1) (6, 7). Differences in gene signature and consequently in functional characteristics exist among DC subsets regarding antigen processing, T-cell stimulatory capacity, and how they respond to pathogens (7, 8). CD8^+^ DCs are efficient in cross-presentation, induction of CD8^+^ cytotoxic T-lymphocyte (CTL) response while CD4^+^ DCs are mainly involved in the activation of CD4^+^ T cells and in the induction and homeostasis of Tregs (5). Moreover, pDCs are the major source of type-I interferon (IFN) and play important role in the induction of antiviral immunity and in regulating the activity of NKs (9, 10). Parallel to the lymphoid organs, three types of DCs are present in most non-lymphoid organs [except the lamina propria and dermis (7, 11)] (Table 1): the CD103^+^DCs (CD45^+^ PDCA-1^−^ CD11c^+^ MHC-II^+^ CD103^+^ CD11b^−^), the CD103^−^DCs (CD45^+^ PDCA-1^−^ CD11c^+^ MHC-II^+^ CD103^−^ CD11b^+^), and pDCs (CD45^+^ CD11c^+^ PDCA-1^+^) (7, 11). The CD103^+^ DCs resemble lymphoid tissue CD8^+^ DCs and can efficiently cross-present cell-associated antigens (7, 11). The CD103^−^ DCs display a heterogeneous population containing cells from both the DC and monocyte lineage (7, 11). Their specific role is less characterized. Of note, non-lymphoid tissues as well as SLOs contain not only fully differentiated DCs but also pre-DC population (CD45^+^ Lin^−^ MHC-II^−^ CD11c^+^) that provide source for DC development and homeostasis *in situ* (12, 13).

**Table 1:** **Murine DC subsets and their role in tolerance**.

	Subgroups	Surface markers	Function in tolerance	Reference
**LOs**				
CD8^+^		CD11c^+^	Induce CD8^+^ T-cell-tolerance	(14–19)
DCs		CD8α^+^ CD4^−^CD11b^−^	Induce *de novo* generation of Foxp3 Tregs	
CD8^−^	CD4^−^ DCs	CD11c^+^ CD8α^−^CD4^+^ 33D1^+^ DC11b^+^	Efficient in activating existent Foxp3 Tregs	(17, 18)
DCs	DN DCs	CD11c^+^ CD8α^−^CD4 CD11b^+^	Unknown	
	→mcDCs	(CD11b^lo/−^)	Presentation of apoptotic cell derived antigens	(20)
	TN DCs	CD11c^+^ CD8α^−^CD4^−^CD11b^−^	Breaking of T-cell tolerance in diabetes	(21)
	Thymic migratory DC	CD11c^+^ CD8α^low^ CD11b^+^ SIRP1α^+^ XCR1^+^	Unknown	
	regDCs	CD11c^lo^ MHCII^lo^ CD11b^hi^ (CD45RB^hi^)	Acquire antigens at the periphery, migrate to the thymus	(22–25)
	L-DCs	CD11c^low^ MHCII^−^DC8α^−^CD11b^hi^	Involved in deletion and Treg induction	
			Production of IL-10 and inducing Tr1 cells and Tregs	(26–31)
			Diminish experimental autoimmune hepatitis	
			Unknown	
pDCs		CD11c^−^MHCII^int^ B220^+^ PDCA-1^+^	Regulate breach of self-tolerance in arthritis	(32–36)
		CCR9^+^ PDCA-1^+^ B220^−^	Induce anergy or deletion of T cells during oral tolerance	
			Aberrant activation promote diabetes and lupus	
			Acquire antigens at the periphery, migrate to the thymus	(22, 24, 37)
			Involved in deletion and Treg induction	
eTACs		CD45^low^, CD11c^low^, MHC-II^hi^, CD357^+^, DC80^int^/86^int^	Induction of tolerance through AIRE-mediated expression of self-antigens	(38)
			Induction of T-cell unresponsiveness of CD4^+^ T cells independent of Tregs	
			Prevention of autoimmune diabetes	
**NON-LOs**				
CD103^+^		CD11c^+^ MHCII^+^ CD11b^−^CD103^+^	Cross-presentation of self-antigens to maintain CD8^+^ T-cell-tolerance	(39–44)
		LP: CD103^+^CD11b^+^	Induce and enhance the *de novo* generation of Foxp3 Tregs	
CD11b^+^		CD11c^+^ MHCII^+^ CD11b^+^ CD103^−^	Need further clarification	
pDC		CD11c^−^B220^+^ PDCA-1^+^	Aberrant activation of pDCs promote diabetes and lupus	(34, 36)

*Arrow indicates that mcDCs belong to the DN DC subset. (CD45RB^hi^) indicates that this marker was investigated and associated with some regDCs only. regDCs, regulatory DCs; DCs, dendritic cells; pDC, plasmacytoid DC; DN/TN DCs, double/triple negative DCs; mcDCs, merocytic DC; eTACs, extrathymic *Aire*-expressing cells; Los, lymphoid organs; non-Los, non-lymphoid organs; LP CD103^+^CD11b^+^, refer to the additional DC subset present in lamina propria besides the CD103^+^ and CD11b^+^ subsets*.

Due to their functional heterogeneity and central spot in antigen presentation, DCs seem to carefully balance between immunity and tolerance. Considering the substantial amount of data available, there are at least five contrasting points to contemplate in order to understand what features describe a tolerogenic DC (tDC) and therefore their influence in autoimmune diseases: (a) Maturation status of DCs, (b) intrinsic characteristics of DCs (involving intracellular signaling, antigen presentation capacity of DCs, and expression of effector molecules), (c) division of labor among DC subsets in tolerance induction, (d) interaction between DCs and other immune or stromal cells, and (e) the effect of the microenvironment to generate DCs with tolerance-inducing potential (e.g., soluble factors).

## Self-Antigen Presentation by DCs: Does DC Maturation Matter?

**Figure 1:**
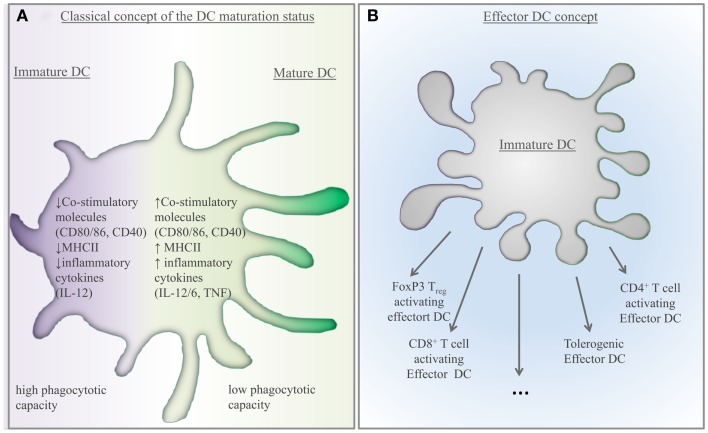
**Scientific concepts: how to characterize tolerogenic DCs? (A) Previous concept described DCs as a cell type existing in two different states: immature and mature DCs**. These categories were based primarily on their co-stimulatory molecule expression, effector cytokine production, and T-cell stimulatory capacity. According to this model, immature DCs were able to induce tolerance. **(B)** Based on novel observations, the existence of multiple effector DCs has been suggested by Reis and Sousa (55). According to this model, immature DCs develop into various types of effector cells. Consequently, effector DCs, capable of inducing tolerance, are the effector tolerogenic DCs.

The early groundbreaking studies have demonstrated in a series of transgenic animal models that cell-associated antigen expressed in peripheral tissues resulted in CD8^+^ T-cell deletion (14, 15). These studies identified DCs as major APCs involved in peripheral tolerance. In these models, DCs acquired cell-associated antigens under non-inflammatory condition from apoptotic cells at the periphery and migratory DCs carried these antigens to the draining lymph node (LN) where CD8^+^ T-cell deletion was initiated (14, 15). This so-called cross-tolerance toward autoantigens involved CD95-signaling (45, 46), Bcl-2 interacting protein (Bim)-dependent apoptosis of T cells (47), and was controlled by cognate CD4^+^ T-cell help (48). The importance of cross-tolerance was additionally demonstrated in an animal model where phagocytosis of apoptotic cells was inhibited in CD11c^+^ cells (16). Transfer of polyclonal CD8^+^ T cells from these animals to Rag1 deficient recipients resulted in an autoimmune phenotype (16). Moreover, viral epitope genetically targeted to CD11c expressing cells caused CD8^+^ T-cell unresponsiveness that was dependent on the engagement of programmed cell death protein-1 (PD1) and cytotoxic T-lymphocyte antigen 4 (CTLA-4) (49). Subsequent studies have similarly demonstrated that model antigen targeted to DCs using C-type lectin receptors (CLRs) such as Dec205 and dendritic cell immunoreceptor (DCIR) induced peripheral CD8^+^ T-cell tolerance and resulted in CD4^+^ Treg induction in the steady state (17, 18). Overall, above data led to the widely accepted notion that immature DCs present self-antigens under non-inflammatory condition and this result in peripheral tolerance. These immature DCs were defined as cells expressing low level of co-stimulatory molecules (CD80, CD86, MHC-II) and failed to produce pro-inflammatory effector molecules such as interleukin (IL)-12 (50) (Figure 1). This notion was underlined by the fact that the same self-antigen presentation by resident DCs using targeting strategy toward, e.g., Dec205, DCIR, or DC NK lectin group receptor-1 (DNGR1) in the presence of anti-CD40 resulted in DC maturation and efficient T helper type 1 (Th1) immunity (17, 18, 51, 52). These mature DCs capable of inducing immunogenic response exhibited high expression of co-stimulatory molecules (CD80, CD86, CD40), upregulated MHC-I and II, and produced pro-inflammatory cytokines such as IL-6, IL-12, and TNF (5) (Figure 1A). Thus, DCs seemed to remain in an immature state during tolerance while they fully mature during induction of immunity. This view was challenged by multiple consecutive studies. CCR7^hi^ MHC-II^hi^ DCs could develop without pathogen within peripheral tissues, after disruption of cell adhesion via E-cadherin and despite their phenotypic maturation; they failed to secrete inflammatory cytokines and elicited a tolerogenic T-cell response *in vivo* (53). Moreover, increasing number of MHC-II^hi^ matured DCs could be observed in draining LN prior to the detection of the autoreactive T and B-cell responses in arthritis (54). Transfer of these matured DCs caused autoimmunity in recipient animals indicating that these cells were responsible for the breaching of self-tolerance (54). Thus, tDCs are not necessarily remaining in an immature state for tolerance induction. Accordingly, it has been suggested by Reis and Sousa that immature DCs could give rise to several different types of “effector” DCs (55). In this model, each type of “effector” DC is functionally distinct and can drive various T-cell responses, such as T helper cell differentiation, induction of CTL, and T-cell tolerance (55). This suggests that tolerance-inducing capacity of DCs is associated with another entity of DCs that is distinct from their immature state (Figure 1B). According to this model, two important questions remain: (i) what features define “effector” DCs with tolerance-inducing capacity and (ii) what signals influence the generation of this “effector” tDC phenotype?

## What Features are Associated with the Tolerogenic Capacity of DCs? – Intrinsic Characteristics of tDCs

### Intracellular signaling events affecting the activation and survival of DCs

There is increasing evidence that tDC phenotype (or development of “effector” tDCs) is an active process and the result of the operation of multiple signaling pathways. In agreement with this, several recent studies have identified key signaling molecules necessary for the tolerogenic function of DCs. One of the prominent pathways involved in this process is the nuclear factor kappa-light-chain-enhancer of activated B cells (NF-κB) pathway. The central role of the NF-κB pathway was demonstrated in DCs specifically lacking A20, a ubiquitin-editing enzyme, which induces the degradation of various signaling molecules that activate NF-κB signaling such as receptor interacting protein-1 (RIP1) (56–59). In these animals, colitis and arthritis developed spontaneously (56). Additionally, milk-fat-globule-EGF VIII (MFG-E8), a secreted molecule that determines the recognition of apoptotic cells, supported the tolerogenic activity of DCs. Mechanistically, MFG-E8 activated signal transducer and activator of transcription 3 (STAT-3) and A20 and decreased pro-inflammatory cytokine production (60) further suggesting the supportive role of the decreased activity of NF-κB pathway in promoting tolerance. Consequently, inhibition of NF-κB and notch homolog 1, translocation-associated (*Drosophila*) (Notch1) by miR-23b promoted tDC differentiation of murine bone marrow dendritic cells (BMDCs) (61). Therefore, over-expression of miR-23b in BMDCs produced less IL-12, increased level of IL-10, and demonstrated enhanced Treg inducing capability *in vitro* (61). NF-κB plays a significant role in DC activation (62) and consequently inhibition of this pathway likely shifts the balance toward tolerance. Surprisingly, unstimulated NF-κB1 deficient DCs pulsed with self-antigen were able to mount CD8^+^ T-cell response and induced autoimmunity (63). This indicates that some degree of activation of this pathway is required for maintaining tolerance as well. It is possible that a combination of pathways will determine whether finally tolerance or immunity occurs and which effector DC phenotype will be the end result of the various stimuli.

Furthermore, p50, active form of NF-κB1, regulated the immunogenicity and life span of DCs (64). According to this, p50 deficient DCs produced higher level of pro-inflammatory cytokines, exhibited increased T-cell stimulatory capacity, and showed longer survival (64). The lifetime of DCs provides an interesting aspect of how tolerance and immunity is regulated and it is thought to be at least partially determined by intrinsic properties of DCs (65). Under physiological condition, DCs die within 48 h after the activating stimuli (66). Significant accumulation of DCs has been observed in MRL-lpr/lpr mice suggesting a connection between apoptosis and autoimmunity (67). Moreover, over-expression of the caspase inhibitor p35 in CD11c^+^ cells resulted in accumulation of DCs and anti-nuclear antibody production in aged mice (65). FAS (68) or Bim deficiency (69) in DCs also caused autoimmunity including autoantibody production. Thus, besides NF-κB, apoptotic pathways regulate the lifetime of DCs and they provide additional checkpoint to maintain tolerance.

Generally, intracellular signaling events, that negatively regulate DC activation, have been implicated to balance tolerance vs. immunity. These pathways primarily act through affecting the size of DC compartment or the extent of the DC activation. Accordingly, DCs deficient in protein tyrosine phosphatase-1 (SHP1) promoted strong Th1 activation that resulted in glomerulonephritis and autoantibody production in aged mice (70). Furthermore, DC-specific deletion of Lyn tyrosine kinase, a negative regulator of the myd88 pathway, resulted in spontaneous T- and B-cell activation, which caused lupus-like autoimmune disease (71). Additionally, STAT-3 deficiency in DCs was also associated with their increased T-cell stimulatory activity and caused ileocolitis resembling human inflammatory bowel disease, suggesting its role in mucosal tolerance (72). Transgenic mice, where suppressor of cytokine signaling-1 (SOCS-1) expressed only in the T- and B-cell compartment exhibited B-cell hyper activation and autoantibody production. SOCS-1 deficient DCs in these animals produced more B-cell activating factor (BAFF), which contributed to the observed autoimmune phenotype (73). Negative regulatory motifs such as immunoreceptor tyrosine-based inhibitory motif (ITIM) containing molecules could affect the numbers and activity of the DCs and thereby tolerance as well. DCIR, a C-type lectin, has been identified as a negative regulator of DC expansion in spleen (74). Consequently, DCIR deficient mice spontaneously developed autoimmune sialadenitis and enthesitis (74).

### Antigen capture, processing, and presentation

Dendritic cells acquire antigens via phagocytosis, receptor mediated endocytosis, and macropinocytosis that lead to the presentation of these antigens to T cells (5). Autoimmune diseases are associated with multiple autoantigens against which the tolerance is broken (75–78). Therefore, the ability of DCs to obtain, process, and present self-antigens is key in understanding tolerance and to closer define the tDC phenotype. Along this line, the antigen-uptake, the nature of antigen, and the specialized machinery associated with tolerance or autoimmunity need to be considered.

Firstly, the mechanism of antigen capture can influence the outcome of the response induced by DCs. Indeed, apoptotic cells (unlike necrotic cells) or soluble proteins, as major sources of self-antigen presentation at the periphery, resulted in tDC activation (50, 79). In case of apoptotic cells, TAM receptor tyrosine kinases (Tyro3, Axl, and Mer) expressed in apoptotic cell membranes triggered SOCS-1 and SOCS-3 expression in DCs, which inhibited the toll like receptor (TLR) and cytokine-induced signaling cascades and therefore the immunogenic DC maturation (80). Underlining this, TAM triple gene (Tyro3, Axl, Mer) deficient mice possessed hyperactive DCs and developed systemic autoimmunity (81). Moreover, the uptake of apoptotic cells triggered transforming growth factor β (TGFβ) release, which led to DC-mediated Treg induction (82, 83). Accordingly, the DC-specific loss of TGFβ activating integrin (αvβ8) resulted in the failure of Treg development initiated by DCs *in vitro* and caused autoimmune colitis *in vivo* (84).

For the uptake of soluble proteins as source of self-antigens, an important antigen-uptake receptor group is the CLRs. They play a role in the uptake of glycosylated antigens. The recognition of most CLRs was not pathogen-restricted, as they often interacted with self-glycoproteins (85–87). Thus, CLRs were involved in the clearance of multiple soluble self-antigens such as thyroglobulin by the mannose receptor (87). In particular, CLRs directed antigen to both MHC-I and MHC class II to prime CD4^+^ and CD8^+^ T-cell responses (88, 89). Targeting antigen to Dec205 or DCIR on DCs is a classical example of inducing antigen specific tolerance toward the antibody coupled soluble protein (17, 18). Additionally, engaging the mannose receptor by mannosylated myelin peptide inhibited EAE (90). Similarly, oral administration of mannose-enriched antigens can induce oral tolerance and favor the generation of IL-10-producing type 1 T regulatory cells (Tr1 cells) via SIGNR1 expressed on DCs of the lamina propria (91). It is less understood which is the exact self-antigen recognition repertoire for each CLRs, and to what extent CLRs on DCs influence autoimmunity. Nevertheless, their intracellular signaling motifs (either ITIM or ITAM motifs) could greatly influence DC activation and effector cytokine production (86) and thereby could influence the tDC phenotype upon antigen capture.

Secondly, in several autoimmune disorders multiple post-translational protein modifications have been observed resulting in alteration of self-antigens and neoantigen formation against which the immune system has not been exposed and tolerized. Multiple autoimmune disorders were dependent on the presence of such post-translational modifications of autoantigens (77). Acetylation of myelin basic protein was required for the development of EAE as non-acetylated peptides failed to stimulate T cells or induce the disease (92, 93). Similar post-translational modifications were involved in the autoimmune process in lupus, celiac disease, and psoriasis (75, 77). Importantly, these modified proteins could be produced and/or taken up by DCs for presentation to T and B cells. So far there is limited understanding of how these modified proteins are captured or produced by DCs, what are the exact consequences of this presentation in disease development, and whether specific DC subsets could be skewed toward presenting these modified proteins during autoimmunity. In line with this, certain modifications such as citrullination could alter the peptide generation of DCs for MHC-II by altering the susceptibility of antigen to cathepsin D (94). On the opposite end of the spectra are the enzymes involved in creating these post-translational modifications. They could affect tolerance such as the *N-*acetyl glucosaminyl transferase (Mgat5) involved in glycosylation process. Mtga5 deficient animals exhibited profound autoimmune disease due to the decreased threshold for T-cell activation (95, 96). Accordingly, increasing N-glycan branching inhibited TCR activation in autoimmune models of EAE and diabetes (97). It will be important for future studies to dissect the involvement of this and similar enzymes in autoimmunity in a cell specific manner especially focusing on DCs.

Thirdly, differences in antigen processing machinery might affect tolerance toward self-antigens. Accordingly, murine CD8^+^ (Dec205^+^) and CD4^+^ (DCIR^+^) DCs differed in their antigen processing machinery, as CD8^+^ DCs were specialized in cross-presentation while CD4^+^ DCs were more potent inducers of CD4^+^ T-cell activation (17). These differences were based on a distinct expression of antigen processing components such as TAPs, cathepsins, and HLA-DM (17).

The proteasome is involved in the production of most MHC-I ligands and therefore considered as main component of the antigen processing machinery (98). Interestingly, autoimmune disorder such as scleroderma was associated with allele variants of immunoproteasome subunits, LMP2 and LMP7 (99). Also, local immunopathology could be explained by tissue specific differences in the proteasomal processing of MHC-I epitopes in a colitis model (100). Additionally, during inflammation the upregulation of LMP7 immunoproteasomal subunit at the periphery was associated with the prevention of diabetes (101). As opposite to this, over-expression of the LMP7 in splenocytes was required for CD8^+^ T-cell auto-reactivity (102). While above studies demonstrate the clear participation of the proteasome in autoimmune processes, it is less understood how cell specific (DC-specific) changes in these components influence disease development. Such cell or subset specific alterations could be especially interesting, as in the thymus, different sets of the proteasome subunits are expressed in mTECs and cTECs suggesting specialization for presentation of self-antigen repertoire for tolerance induction (103). In scleroderma, DC-specific alteration in proteasomal processing was associated with the disease (76, 104). In this case, the unusual processing of topoisomerase-I by the nucleoproteasome in DCs was connected with autoantibody production and clinical manifestation of this autoimmune disorder (76, 104).

The proteasome generates peptides some of which are further trimmed by aminopeptidases. Some of the trimming takes place in the cytoplasm but a large proportion is located within the endoplasmic reticulum (ER). One of the primary enzymes in the ER is the ER associated aminopeptidase (ERAP) (105). These trimming enzymes in humans were associated with susceptibility to various autoimmune diseases (106). For example, based on genetic studies, ERAP1 was highly associated with ankylosis spondylitis (107) and ERAP2 was linked to Crohn’s disease (108). Whether specific alterations in such peptide processing are associated explicitly with DCs needs further evaluation.

In terms of the presence of specialized intracellular compartments associated with tolerance or autoimmunity, merocytic DCs (mcDCs) that were able to breach self-tolerance (20)(Table 1) possessed specialized vesicles where they could store apoptotic cellular material for autoantigen presentation for an extended period of time. Nevertheless, the understanding of these intracellular organelles is limited so far.

Hence, it remains to be further explored whether altered antigen presentation machinery exists and would be associated with DCs inducing tolerance and/or with DCs breaching tolerance.

Taken together, various signaling pathways and processes influencing antigen handling and processing determine the capacity of DCs for tolerance induction and dysregulation in these pathways could result in alteration of tDC “effector” phenotype toward promoting autoimmunity.

## What Features Define tDCs? – Effector Characteristics of tDCs

The tolerogenic effector capacity of DCs predominantly has been analyzed in functional co-culture assays (induction of Tregs or Tr1 cells), determining how DC-transfer affected disease outcome or via using transgenic animal models (3, 50). Moreover, increased expression of IL-10 or TGFβ and reduced expression of pro-inflammatory cytokines (e.g., IL-12, IL-1, IL-6, TNF) and co-stimulatory molecules (e.g., CD80, CD86) are typically considered as hallmark of tDCs (3, 50) (Figure 1B). In NOD mice, DC-derived IL-2 was required for CD8^+^ T-cell deletion and for protection from diabetes (109). Additionally, DC-derived IL-2 together with CD40–CD40L interaction were involved in Treg homeostasis (110–112) establishing IL-2 as novel effector molecule for tDCs. Besides, variety of enzymes such as retinaldehyde dehydrogenase-2 (RALDH2) involved in retinoid acid (RA) metabolism and indolamine 2,3 dioxygenase (IDO) altering tryptophan metabolism were associated with tDCs (3, 113). RA was involved in Treg induction primarily in the gut and skin while IDO could inhibit the proliferation of activated T cells and enhanced the induction of Tregs (3, 113). It has been recently demonstrated that the non-enzymatic activity of IDO upon TGFβ challenge in pDCs was involved in maintaining their regulatory phenotype (114). IDO mediated intracellular signaling in pDCs, evoked the capacity of these cells to suppress Th1 immunity, and resulted in increased Treg differentiation *in vivo* (114).

Apart from this, there is increasing evidence suggesting a high level of complexity associated with the tDC “effector” phenotype. The effect of dexamethasone and vitamin D on human DCs has been recently characterized at a molecular level (115). Both compounds alone and in combination induced tDCs and have been widely used to generate these cells *in vitro* (113). Interestingly, the tDC phenotype was associated with unique protein profiles with severe impact on metabolic pathways (115). These pathways affected lipid, glucose, and oxidative phosphorylation in tDCs. Moreover, they altered the production of ROS, the survival of DCs, and the dependence of DCs on available nutrients (115). This is in line with the observation that after TLR stimuli, the metabolic status of DCs transitioned from oxidative phosphorylation to glycolysis (116). This transition was partially inhibited by IL-10, a cytokine associated with tolerance (116). Hence, it is likely that the tolerogenic potential of DCs is associated with a specific metabolic fingerprint that supports DC function in maintaining immune homeostasis. Also, blocking mammalian target of rapamycin (mTOR) signaling via rapamycin during DC maturation resulted in tDCs, which promoted alloantigen specific tolerance (117). Although, mTOR affects multiple cellular processes and only one aspect of them is associated with metabolism, further studies are needed to clarify whether the above effect of rapamycin was due to specific metabolic changes associated with tDCs.

Overall, it seems that the specific features of “effector” tDCs are more complex than previously thought. The broader determination of the switch in metabolic status, the checkpoints regulating this change together with the intracellular pathways, and secretome profile of tDCs might provide more precise specifications of what the tDC phenotype means. It is plausible that these features are slightly different dependent on the microenvironmental factors affecting DCs and might show organ or even DC subset specific amendments.

## Division of Labor for Tolerance Induction

Various animal models have demonstrated the importance of CD11c^+^ cells in the maintenance of tolerance (3, 14–18, 49, 50). Supporting this notion, transient depletion of CD11c^+/hi^ cells aggravated immune pathology and inflammation (118). Rather surprisingly, the constitutive ablation of CD11c^+^ cells showed myeloproliferative disorder associated with elevated serum Fms-like tyrosine kinase-3 ligand (FLT3L) level (119, 120). Between the two pioneering studies on constitutive DC depletion, only Ohnmacht et al. found impaired negative selection of CD4^+^ T cells and the development of inflammatory bowel disease (120). Although the ultimate role of DCs in autoimmunity could not be demonstrated in these studies, they pinpointed an important regulatory circuit within the myeloid cell compartment. Importantly, unlike CD11c, novel molecules such as the transcription factor zinc finger and BTB domain containing 46 (zbtb46) and DNGR1 were exclusively expressed by DCs and were absent in NK cells, pDCs, or monocytes (121, 122). It will be interesting to investigate in future studies how short- or long-term depletion of DCs using the above-mentioned markers would affect autoimmunity and peripheral tolerance.

Given the heterogeneity of DCs, genetic models where certain DC subtype was missing provided interesting insight into the process associated with immune homeostasis. *Batf3* deficient mice lack CD8^+^ DCs in SLOs and CD103^+^ DCs at the periphery (123). Despite this loss in these subtypes, the mice under steady state have no obvious autoimmune phenotype (123). Nonetheless, renal LN CD8^+^ XCR1^+^ DCs were absent in *Batf3* deficient animals and therefore failed to induce tolerance against soluble antigen concentrated in the kidney (19). Additionally, pulmonary tolerance toward inhaled antigen correlated with the ability of CD103^+^ DCs to upregulate RALDH2, which promoted forkhead box P3 (FoxP3) expression in Tregs (39). Correspondingly, *Batf3* deficient mice failed to induce tolerance toward inhaled antigen (39). Besides the lung, the CD103^+^ DC subpopulation in the gut prevented colitis and was efficient in inducing Tregs via production of TGFβ, RA, and induction of IDO (40–42). Similarly, CD103^+^ skin migratory DCs were responsible for tolerance induction by transporting skin-associated antigens into draining LNs (43). Since *Batf3* deficient animals showed no obvious autoimmune phenotype, it is likely that other subsets took over the tolerance-inducing function of the missing DC subtype.

Also, pDCs have been identified as guardians of immune homeostasis in arthritis (32) and oral tolerance (33). Despite these data, transient depletion of pDCs did not result in spontaneous autoimmune disorder (10, 124). Additionally, pDCs have been associated with multiple autoimmune disorders (34–36). Thus, the question is whether do individual subsets of DCs specialized in tolerance exist and is there functional redundancy among the DC subsets?

A novel subset of tDCs has been recently identified within SLOs localized at the T–B cell border: the extrathymic Aire-expressing cells (eTACs) (38). These cells were CD45^lo^CD11c^lo^ and positive for zbtb46, therefore could be identified as DCs (38). Besides, eTACs expressed high level of Epcam and MHC-II but low level of co-stimulatory molecules (Table 1). Importantly, eTACs functionally inactivated autoreactive CD4^+^ T cells independent from Tregs and were unresponsive to a variety of inflammatory stimuli (38).

Moreover, Wakkach et al. have distinguished in the spleen, the IL-10 secreting tDCs harnessing CD11c^lo^CD45RB^hi^ surface markers (Table 1) (26). These cells showed immature phenotype and induced the differentiation of Tr1 cells (26). Additionally, they were resistant to various inflammatory maturation stimuli and upon adoptive transfer they induced antigen-specific unresponsiveness in recipient mice (26). Further studies demonstrated that the differentiation of CD11c^lo^CD45RB^hi^ tDCs from hematopoietic precursors could be instructed by splenic stromal cells (27) and via utilizing neuropeptides such as vasoactive intestinal peptide (VIP) and pituitary adenylate cyclase-activating polypeptide (PACAP) (125).

Specific peripheral DC populations could migrate via blood to the thymus and contributed to central tolerance. Approximately 50% of the thymic DCs arrived from the peripheral blood and represented the migratory DC population in this organ (126). This migratory DC population in the murine system consisted of CD11c^+^CD8α^low^CD11b^+^SIRP1α^+^ conventional DCs, CCR9^+^ pDCs, and distinguished from the resident thymic DC population (CD11c^+^CD8α^hi^CD11b^−^SIRP1α^−^) (22–24, 37, 126, 127) (Table 1). Importantly, similar DC subsets were described in humans as well (128). The three murine DC subsets differed in their thymic localization, chemokine receptor requirement for their intrathymic positioning, and their origin (25, 37, 129). The migratory thymic DC populations are especially interesting for tolerance induction. SIRP1α^+^ DCs and pDCs sampled blood borne antigens and transported them to the thymic cortex area where they contributed to clonal deletion and Treg induction (23, 24, 127). Additionally, SIRP1α^+^ DCs has been implicated in negative selection toward circulating tumor antigens thereby promoting tumor tolerance (130). Moreover, in an experimental system where model antigen was expressed in cardiac myocytes in a membrane-bound form, autoantigen presentation depended on VLA4-mediated recruitment of migratory peripheral DCs to the thymus (22) suggesting that cell-associated antigen was transported by migratory DCs to the thymus. Also, pDCs could acquire particulate antigens injected subcutaneously from the skin and transported to the thymus for tolerance induction (37). Regardless, it remains to be elucidated how these DCs sample antigens from peripheral organs before migrating to the thymus, what is their exclusive physiological contribution in tolerance induction, and what regulatory circuits play a role in their migration. Interestingly, TLR ligands downregulated the capacity of these DCs to reach the thymus (22, 37), thus separating the immunogenic response toward pathogens from the thymic tolerance. Another intriguing possibility about these cells is that they may transport antigens from the digestive tract that could potentially result in tolerance toward food-related antigens (103). This possibility needs further investigation in the future.

Importantly, in balancing tolerance and immunity, tDCs represent one side of the spectrum and on the other side are the DC subtypes, which are specifically promoting autoimmunity. Such DC subtype has been also found in NOD mice (20). This subtype of DCs is called mcDCs (Table 1). The frequency of mcDCs was elevated in spleen and pancreatic LNs of NOD mice possessing insulitis (20). Importantly, these cells could acquire apoptotic cellular materials and induce T-cell activation that reversed the deletion of self-reactive T cells (20). Moreover, upon transfer to young NOD recipients, antigen loaded mcDCs could break peripheral tolerance toward β-cell antigens (20). The number of mcDCs within the spleen was negatively regulated by the Idd13 locus which was previously associated with diabetes prevention (21). It remains to be elucidated whether mcDCs could break tolerance toward other antigens than β-cell related ones *in vivo* thus indicating a general tolerance breaking DC subtype.

Long-term culture of splenic stromal and hematopoietic cells could also result in the generation of a novel DC subtype, the L-DCs (Table 1). L-DCs were superior in cross-presentation of soluble antigens *in vitro* compared to CD8^+^ DCs (131). Interestingly, adoptive transfer of these DCs induced immunogenic CD8^+^ T-cell activation *in vivo* (131). It will be interesting to see whether according to their immunogenic properties they could manifest DC subtypes breaching CD8^+^ T-cell tolerance.

Future studies should illuminate whether there might be a functional cross-talk among tDCs, the tolerance breaching DC population, and the well-established lymphoid organ resident DCs during prevention and development of autoimmune disease.

## Interactions between tDCs and Immune or Stromal Cells for Tolerance Induction

Dendritic cells were capable of inducing or activating Tregs in multiple ways (3, 113). The rather surprising discovery was the participation of DCs in maintaining homeostasis of Tregs. According to this, transient depletion of CD11c^+^ cells reduced the frequency of Tregs (118) while the expansion of DCs using FLT3L resulted in increased Treg numbers *in vivo* (118, 132, 133). Moreover, MHC-II expression by DCs was required to maintain the Treg population at the periphery (118). These results suggested that the DC-Treg feedback would set the tone for tolerance. Accordingly, human diabetes patients displayed lower DC numbers than healthy ones (134). Also the expansion of Tregs, due to increased DC numbers, reduced severity of colitis and arthritis (118, 133, 135). Despite these data, in some cases autoimmunity was associated with increased number of DCs, but was not accompanied with altered Treg numbers (65, 74). This could indicate additional factors, which might influence Treg homeostasis or could pinpoint functional alterations of the expanding DC population. Indeed, recent study demonstrated that DCs generated using FLT3L lacked the ability to induce Tregs *in vitro* (136). Moreover, the expansion of donor liver DCs, using FLT3L before transplantation, abrogated liver allograft acceptance and resulted in graft rejection (137). The discrepancies in the effect of FLT3L as well as the exact relation in DC and Treg numbers *in vivo* remain to be determined.

Another type of cellular cross-talk between Tregs and DCs has been recently demonstrated in the murine model of contact hypersensitivity (138). Here Tregs conditioned DCs to induce regulatory CD8^+^ T cells that could protect against the disease (138). how and what is the molecular mechanism of this imprinting needs further clarification.

Autoimmune arthritis induced in B-cell deficient mice resulted in exacerbation of the inflammatory response. In this model, DCs produced higher amount of inflammatory cytokines due to the missing control by the IL-10^hi^ B-cell subpopulation (139). Similar phenomenon exists between human B cells and DCs, where B cells in soluble and cell contact dependent manner regulated DC activation and IL-12 production (140). This suggests a close interplay between these two cell types while maintaining homeostasis.

Not only B cells but also innate cells such as NKT could control tDC function. Treatment of NOD mice with NKT activating ligand such as α-galactosyl ceramide resulted in the accumulation of tDCs in draining LN (141). These tDCs anergized autoreactive T cells and therefore prevented diabetes (141). The interaction between NKT cells and DCs was rather complex, bidirectional, and not restricted to only tolerogenic outcome (142, 143). The exact circumstances when NKT cells act toward the development of tDCs remain to be elucidated.

In the last few years, multiple studies have demonstrated that LN stromal cells are capable of inducing T-cell tolerance (144–147). Anatomically, stromal cells within SLOs are positioned in close proximity with lymphoid resident DCs (148) and guide migratory DCs within SLOs (149). In addition to this, stromal cells inhibited the capacity of DCs to activate T cells (150, 151). Furthermore, they prompted hematopoietic progenitors to differentiate toward regulatory IL-10 producing tDCs (27). During *Leishmania* infection, splenic stromal cells upregulated chemokine (C–X–C motif) ligand 12 (CXCL12) and CCL8 to specifically attract hematopoietic precursors and induced tDC differentiation *in situ* (152). Importantly, tDCs have been identified under steady state in lung (28), spleen (26, 27, 29), and liver (30). Moreover, stromal cells directed the differentiation of not only hematopoietic precursors but also matured DCs toward regulatory ones. These tDCs produced nitric oxide and IL-10 and consequently dampened T-cell responses (31). Importantly, in adoptive transfer experiment, the tDCs promoted by liver stromal cells diminished experimental autoimmune hepatitis (30). Thus, it is likely that these DCs not only provide important negative regulatory circuit during T-cell activation but also contribute to maintain tolerance. It still needs to be clarified what is the exact role of these DCs under steady state and whether they could play a role in balancing autoimmunity and tolerance.

## Which Soluble Molecules Endorse the tDC Phenotype?

The soluble molecules involved in inducing tDC phenotype can be generally divided into two groups: the ones which promote differentiation of tDCs from hematopoietic precursors or peripheral blood monocytes and the ones that directly act on immature DCs (113, 153). Not only natural biomolecules but also multiple pharmacological compounds have been used to generate tDCs *in vitro* (113, 153). These experiments generally combined basic differentiation factors, such as granulocyte macrophage-colony-stimulating factor (GM-CSF) for murine BMDCs, with the variety of soluble molecules and characterized the tolerogenic phenotype of the developed DCs *in vitro* (113, 153). IL-10, TGFβ, TNF, IL-6, hepatocyte growth factor, prostaglandins, and vitamin D were identified as effective molecules in inducing tDC phenotype *in vitro* (113). Hormones could also affect DC maturation and the tolerance-inducing competence of DCs. In particular, glucocorticoids suppressed DC maturation and generated tDCs *in vitro*. Glucocorticoids acted via nuclear receptors followed by the induction of glucocorticoid induced leucine zipper (GILZ) (154). GILZ is a transcription factor, which was absolutely required for glucocorticoid-mediated tDC differentiation (154). DC-specific transcript (DC-SCRIPT), a corepressor of GILZ has been recently identified in DCs (155), indicating a network of transcription factors that counterbalances the effect of glucocorticoids in immunity vs. tolerance. It remains to be identified whether changing the balance of these transcription factors can be used as therapeutic target for generating tDCs for therapy as well.

Although most of the above-mentioned compounds were used in a combination with GM-CSF *in vitro*, the effect of GM-CSF itself in tolerance is not straightforward. GM-CSF deficient animals developed lupus-like systemic autoimmune disorder and GM-CSF together with IL-3 promoted diabetes (156, 157). On the other hand, in the absence of GM-CSF, mice were protected against collagen-induced arthritis (158).

Pro-inflammatory mediators such as IFNγ and TNF could transform DCs into inhibitory IDO expressing tDCs. Such IDO^+^ DCs induced oral tolerance and prevented arthritis and colitis (41, 159). IDO expression and induction of tDCs could be initiated by chemokine (C–C motif) ligand 18 (CCL18) as well (160). The role of cytokines affecting DC function under steady state could be of relevance, as these molecules could actively maintain the tolerogenic environment. This could be underlined by the fact that asthmatic patient exhibited reduced CCL18 binding to its receptor suggesting a protective role of CCL18 under steady state (160). As opposite to this, cytokine signaling could also contribute to the breaking of tolerance. Indeed, IL-1R1 signaling in DCs promoted autoreactive CD4^+^ T-cell expansion and caused autoimmune myocarditis (161).

There are increasing examples of novel soluble molecules, with known primary function unrelated to DC biology that can incite immature DCs with a tolerogenic capability. Adiponectin, which is an adipocytokine with anti-inflammatory properties, increased programmed cell death 1 ligand (PDL-1) expression of DCs and thereby intensified their Treg inducing capacity (162). Likewise, adiponectin deficient mice exhibited severe cardiac transplant rejection (163). Further studies are required to delineate its effect in tolerance induction.

Thrombomodulin (TM), a cofactor of thrombin, turned BMDCs to secret IL-10 independent of its thrombin and coagulation related function (164). Importantly, transfer of TM^+^ DCs protected recipient animals against airway hypersensitivity (164). Another novel molecule involved in DC biology is adrenomedullin, a calcitonin related neuropeptide. This molecule induced IDO in immature BMDCs and thereby promoted the conversion of CD4^+^ T cells to CD4^+^ CD25^+^ Foxp3^hi^ Tregs *in vitro* (165).

Additionally, it has been also recently identified that Wnt3a and Wnt5a directly induced immunoregulatory cytokine expression by DCs and promoted Treg development. Interestingly, Wnt3a acted via β-catenin signaling while Wnt5a triggered other signaling pathways (44, 166). β-Catenin signaling in intestinal DCs induced RALDH2, inhibited the expression of pro-inflammatory cytokines, and promoted their Treg inducing capacity (44).

In mucosal sites, secretory IgA encompasses protective role against invasion of various pathogens but it seems that it exhibits further functions within the circulation. SIGNR1 binding to secretory IgA on BMDCs rendered these cells resistant to TLR dependent maturation (167). IgA primed BMDCs showed higher capacity to induce Tregs via their IL-10 production and were able to inhibit autoimmunity in animal models of diabetes and EAE (167).

Taken together, a long line of biomolecules is available with the capacity to either alter DC function or promote tDC differentiation. It is not clear yet whether all *in vitro* defined tolerogenic signals truly induce similar DC activation *in vivo* or other factors might intervene with their effect *in vivo*. Most of these soluble molecules are in the focus of tDC research to utilize them for generating human tDCs from autologous bone marrow or from peripheral blood monocytes. Autologous transfer of tDCs has been tested in clinical trials and was well tolerated in diabetic patients (168, 169). Although this approach provides attractive therapeutic possibilities, more research is needed to evaluate and understand the complexity of tolerance, such as the stability of the tDC phenotype *in vivo* and the dose and route of tDC vaccine used for the treatments of autoimmune patients.

## Summary and Conclusion

**Figure 2:**
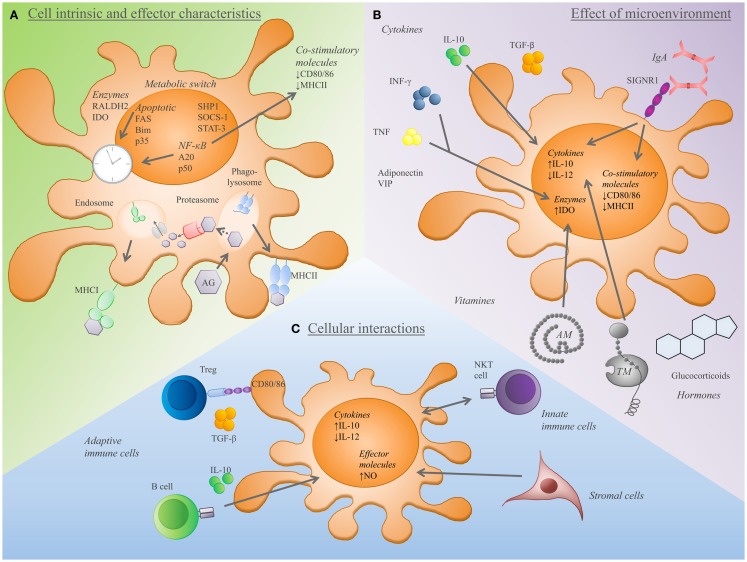
**Components that determine the establishment and tolerance-inducing capacity of tolerogenic effector DCs**. **(A)** DCs expressing high level of anti-inflammatory cytokines (IL-10, TGFβ) and low level of co-stimulatory molecules (DC80/86) show a tolerogenic rather than an immunogenic phenotype. Additionally, the capacity of DCs to express Raldh2 or IDO is associated with tolerance. Furthermore, the activity of several pathways is linked to tDCs, such as metabolic, apoptosis, and NF-κB pathway, or activity of SHP1 and STAT-3. Additionally, the antigen capture and processing machinery (uptake of apoptotic cells, antigen-uptake receptors such as CLRs together with the MHCI and II processing machinery) greatly influence the T-cell inducing and tolerogenic capacity of DCs. **(B)** A variety of biological substances have an impact on tDC differentiation and function. Cytokines, vitamins, hormones as well as antibodies, thrombomodulin (TM), adrenomedullin (AM), and VIP induce tDCs. **(C)** The dialog of DCs with other immune cells and stromal cells provides additional checkpoints for the maintenance of tolerance. DC-Treg crosstalk involves the regulation of Treg homeostasis, the activation and induction of Tregs. Tregs, IL-10 expressing B cells, and natural killer T (NKT) cells favor a tDC phenotype. In addition, stromal cells promote tDC differentiation toward IL-10 or nitric oxide (NO) producing regulatory tDCs.

In peripheral tolerance, similarly to the thymus, various APCs are involved to guard immune homeostasis. DCs are the major APCs involved in this process. Multiple components are implicated in maintenance and/or induction of tolerogenic effector DC phenotype (Figure 2). Intrinsic signaling and antigen processing properties of DCs together with the impact of the microenvironment influence the tolerogenic adeptness of DCs. It is itself intriguing that a variety of active processes seem to be necessary for mediating immune homeostasis and it is clearly not a passive effect of the missing maturation signal as previously thought. The picture is further complicated with the fact that various subtypes of DCs seem to possess different capacity for tolerance. Similarly in immunity, sequential antigen presentation by different DC types resulted in different aspects of T-cell activation and effector differentiation (170). Thus, maintenance of immune homeostasis is a result of a complex interaction of soluble and cell-associated components. Understanding this network and thereby influencing DCs provide important targets for treatment of autoimmune disease.

## Author Contributions

Ann-Katrin Hopp contributed with literature search, prepared the table and the figure and figure legend, and critically read the manuscript. Anne Rupp contributed with literature search. Veronika Lukacs-Kornek developed the concept of the manuscript, supervised, and wrote the manuscript.

## Conflict of Interest Statement

The authors declare that the research was conducted in the absence of any commercial or financial relationships that could be construed as a potential conflict of interest.

## Abbreviations

Batf3, basic leucine zipper transcription factor, ATF-like 3; Bim, Bcl-2 interacting protein; CCR7, chemokine (C–C motif) receptor 7; CCR9, chemokine (C–C motif) receptor 9; CCL18, chemokine (C–C motif) ligand 18; CTL, cytotoxic T-lymphocyte; CTLA-4, cytotoxic T-lymphocyte antigen 4; CXCL12, chemokine (C–X–C motif) ligand 12; Flt3L, FMS-like tyrosine kinase-3 ligand; FoxP3, forkhead box P3; GM-CSF, granulocyte macrophage-colony-stimulating factor; IFN, interferon; IL, interleukin; MHC-II, major histocompatibility complex II; LPM2 and 7, proteasome beta subunits; NF-κB, nuclear factor kappa-light-chain-enhancer of activated B cells; Notch1, notch homolog 1, translocation-associated (*Drosophila*); PD1, programmed cell death protein-1; PDL-1, programmed cell death 1 ligand; SIRP1α, signal regulatory protein-1 alpha; STAT-3, signal transducer and activator of transcription 3; TGFβ, transforming growth factor β; Th1, T helper type 1; TLR, toll like receptor; TNFα, tumor necrosis factor-alpha; Tr1 cells, type 1 T regulatory cells; zbtb46, zinc finger and BTB domain containing 46; XCR1, chemokine (C motif) receptor-1.
